# A Linear Predictor Based on FTIR Spectral Biomarkers Improves Disease Diagnosis Classification: An Application to Multiple Sclerosis

**DOI:** 10.3390/jpm13111596

**Published:** 2023-11-11

**Authors:** Francesca Condino, Maria Caterina Crocco, Domenico Pirritano, Alfredo Petrone, Francesco Del Giudice, Rita Guzzi

**Affiliations:** 1 Department of Economics, Statistics and Finance ”Giovanni Anania”, University of Calabria, 87036 Rende, Italy; francesca.condino@unical.it; 2 STAR Research Infrastructure, University of Calabria, 87036 Rende, Italy; mariacaterina.crocco@unical.it; 3 Department of Physics, Molecular Biophysics Laboratory, University of Calabria, 87036 Rende, Italy; 4 SOC Neurologia, Azienda Ospedaliero-Universitaria Renato Dulbecco, 88100 Catanzaro, Italy; pirritanodomenico@gmail.com; 5 UOC Neurologia, Azienda Ospedaliera dell’Annunziata, 87100 Cosenza, Italy; a.petrone@aocs.it (A.P.); fra.delgiu@libero.it (F.D.G.); 6 SOC Neurologia, Ospedale Jazzolino, Azienda Ospedaliera Provinciale, 89900 Vibo Valentia, Italy; 7 CNR-NANOTEC, Department of Physics, University of Calabria, 87036 Rende, Italy

**Keywords:** multiple sclerosis, plasma, ATR-FTIR, multivariate analysis, discrimination, logistic regression

## Abstract

Multiple sclerosis (MS) is a neurodegenerative disease of the central nervous system that can lead to long-term disability. The diagnosis of MS is not simple and requires many instrumental and clinical tests. Sampling easily collected biofluids using spectroscopic approaches is becoming of increasing interest in the medical field to integrate and improve diagnostic procedures. Here we present a statistical approach where we combine a number of spectral biomarkers derived from the ATR-FTIR spectra of blood plasma samples of healthy control subjects and MS patients, to obtain a linear predictor useful for discriminating between the two groups of individuals. This predictor provides a simple tool in which the contribution of different molecular components is summarized and, as a result, the sensitivity (80%) and specificity (93%) of the identification are significantly improved compared to those obtained with typical classification algorithms. The strategy proposed can be very helpful when applied to the diagnosis of diseases whose presence is reflected in a minimal way in the analyzed biofluids (blood and its derivatives), as it is for MS as well as for other neurological disorders.

## 1. Introduction

Multiple sclerosis (MS) is an immune-mediated demyelinating and inflammation disorder of the central nervous system (CNS) and is the major cause of non-traumatic neurological disability in young adults. The latest update on the prevalence of the disease reports that there are about 2.9 million people living with MS worldwide, with a higher prevalence in higher income areas [1,2]. Risk factors are not well defined and involve interplay between genetic, lifestyle and environmental factors [3]. There is a gender prevalence in MS, and women are more affected than men in a 3 to 1 ratio. An important feature of this disease is that the clinical course can be highly variable and heterogeneous in the type of clinical manifestations, course of the disease, and degree of disability [4]. The disability level is generally quantified via the expanded disability status scale (EDSS), a numeric index which ranges from 0 (normal status) to 10, with a variation step of 0.5. It is assigned by a neurologist using an empirical assessment, and evaluates neurological findings in eight functional systems, as well as the walking ability of the patient [5]. 

MS diagnosis is not very easy, as the initial symptoms of the disease may be generic and nonspecific, including changes in vision, mobility, and cognitive abilities [6]. There is no single test for the diagnosis, but diagnostic criteria have been validated (namely, the McDonald criteria revised in 2017), based on clinical observation of the patient, as well as laboratory and instrumental tests such as magnetic resonance imaging and oligoclonal bands in the cerebrospinal fluid (CSF) [7]. In particular, evidence of neurological damage disseminated in time and space in multiple regions of the CNS should be seen. Blood tests are generally used to rule out diseases that resemble MS and might be confused with it. The overall protocol requires long time for the tests and evaluations to take place, which are certainly not pleasant for the patients. The ultimate goal is to make an early and accurate diagnosis in order to provide effective drug treatment to control the disease, which currently remains incurable. 

Recently, the application of analytical and spectroscopic methods to analyze human biofluids (e.g., plasma, serum, saliva, urine, bile), searching for biomarkers or even aiming at early stage disease detection, is becoming an important issue in the biomedical research field. Such methods include nuclear magnetic resonance (NMR) [8,9], mass spectrometry [10], differential scanning calorimetry [11,12,13], and Raman and infrared spectroscopy [14,15,16]. These experimental methods are often supported by a number of multivariate statistical approaches in the analysis of their data. Vibrational spectroscopy can be applied in different modalities and has shown great potential to classify, either as healthy or pathological, a number of biological materials, such as cells, tissues, blood, saliva, and for many different diseases [17]. The samples’ preparation requires minimal or no treatment, and experiments are rapid and label-free. These advantages have contributed to a wide range of applications, and studies for diagnostic purposes have been reported for different forms of cancer [18,19], COVID-19 [20,21,22], systemic amyloidosis [23], as well as neurodegenerative diseases [24,25]. Despite infrared spectroscopy having demonstrated its potential as a diagnostic tool, the translation of these experimental and computational techniques into clinical practice does not seem so straightforward. Standardized protocols should be implemented and established at different steps, including sample acquisition and storage, condition measurements, data correction, and analysis [17,26,27]. 

In a previous study, we applied attenuated total reflection Fourier transform IR (ATR-FTIR) spectroscopy to the plasma of 85 subjects: 45 MS patients with different clinical records in terms of disability level and time duration of the disease and 40 healthy controls (HC) with no inflammatory or autoimmune disease diagnosis [28]. Although CSF may be the ideal source of biomarkers for MS, it is not an easy biofluid to be collected, and the availability of healthy samples for comparison is also a limiting factor. In contrast, blood can be routinely collected; it perfuses all body organs, and also exchanges molecules (potential biomarkers) with the CSF. 

The analysis of our experimental data showed that the spectral features of absorbance spectra for HC and MS are similar to each other, with the latter having a slightly higher variability [28]. Using univariate and multivariate statistical approaches on the ATR-FTIR spectra, it was found that: i) MS patients were characterized by a higher lipid/protein ratio compared to HC individuals; and ii) a random forest algorithm was able to select regions for discriminating the disease occurrence with a good degree of accuracy (sensitivity 78%; specificity 83%) with respect to other two classification algorithms (PCA-LDA and PLS-DA).

In this work, we have proposed a new data analysis based on a linear combination of a number of spectral biomarkers, derived from those experimental spectra, including some previously selected. We showed that the predictive model obtained with this approach significantly improves the performance of the classification, especially for the specificity index. The advantage of using this model is that it combines the most important spectral indicators that jointly contribute to the samples’ classification, through a simple expression giving a single score value, and can be easily used to establish whether the subject may likely belong to the class of healthy or diseased individuals. 

## 2. Materials and Methods

### 2.1. Biological Samples and Spectral Acquisition

Blood samples of 45 MS patients with a confirmed diagnosis according to the McDonald criteria [7] were collected at the Annunziata Hospital in Cosenza (Italy). The criteria of inclusion and the degree of disease progression were established by the neurologist. A more detailed description of the clinical and personal information of the patients can be found in ref. [28]. Briefly, the data included: age (average 42.7, range 22–69), gender (31 females, 14 males), disease phenotype/form (38 relapsing-remitting multiple sclerosis, RRMS; 7 secondary-progressive multiple sclerosis, SPMS), disease duration (from 1 to 47 years), disability degree expressed in terms of EDSS (range 0.5–7.0). Most of the MS patients (32/45, 71%) had a mild score (EDSS ≤ 3.0) and the others (13/45, 29%) had a moderate/severe level of disease (EDSS ˃ 3.5). 

HC subjects (n. 40) were recruited at the same Annunziata Hospital (n. 21) and among the blood donors at the Health Center of the University of Calabria (n. 19). The gender distribution was 21 females and 19 males, with an average age of 37.3 years (range 24–60 years, one outlier of 71 years). For all of them there was no evidence of neurological or inflammatory disease. 

All the participants were fully informed about the study and provided their written consent. The study was approved by the Ethics Committee of the northern area of the Calabria region (protocol code n. 50, 14 February 2017).

ATR-FTIR experiments were performed as reported in ref. [28]. For each plasma sample, up to five replicate spectra were acquired and then averaged to obtain the absorbance spectrum of each individual. The regions selected for the statistical data analysis were the fingerprint region (1800–900 cm^−1^) and high region (3050–2800 cm^−1^). 

### 2.2. Computational Analysis

Statistical differences in the vector normalized absorbance spectra of MS and HC groups were assessed using a two-tailed parametric *t*-test. A *p*-value ≤ 0.05 was considered significant in all statistical tests. 

A number of spectral indicators were selected from the absorbance spectra based on their biological significance in this specific study, and also considering those commonly identified in the FTIR spectra of biofluids [25,28,29,30,31]. The considered spectral biomarkers are listed in Table 1, and are used to construct the final linear predictor for distinguishing between healthy controls and patients. To this end, a generalized linear model (see, for example, the data analysis of Agresti [32]) is considered to relate the *p* spectral biomarkers $x=\left(x_{1},\ldots ,x_{p}\right)$ to the probability π(x) of having the disease. In particular, a logit link function is considered, as the response is a binary variable, and the resulting logistic regression model can be expressed as follows:

(1) $$\pi \left(x\right)=\frac{exp\left(\beta_{0}+\sum_{j=1}^{p}\beta_{j}x_{j}\right)}{1+exp\left(\beta_{0}+\sum_{j=1}^{p}\beta_{j}x_{j}\right)}$$

or, alternatively:

(2) $$\mathrm{logit}\left[\pi \left(x\right)\right]=\beta_{0}+\overset{p}{\underset{j=1}{\sum }}\beta_{j}x_{j}$$

where $\beta_{0}$ represents the intercept of the model.

To select the relevant covariates, a backward stepwise selection approach was used for removing the weakest related variables, based on the probability of the Wald statistic. The maximum likelihood estimation method was considered to obtain the regression coefficients, $\beta_{j}\;\left(j=0,\ldots p\right)$, and the Wald test was used to assess their significance.

The obtained estimated linear predictor can be used to classify each subject as diseased or healthy, according to whether it assumes a positive or a negative value or, equivalently, whether the corresponding estimated probability is higher or lower than the cutoff of 0.5. 

The performance of the final model in predicting MS diagnosis was evaluated through the usual metrics, namely sensitivity, specificity, and accuracy. Furthermore, the receiver operating characteristic (ROC) curve was obtained and the area under the curve (AUC) is considered a measure of the predictive power of the method. Finally, a bootstrap method was used to test the difference in the AUC of the ROC curves among patients with different EDSS scores or disease duration. 

## 3. Results

### 3.1. ATR-FTIR Spectra of MS and HC Plasma Samples: Identification of the Spectral Biomarkers

**Figure 1:**
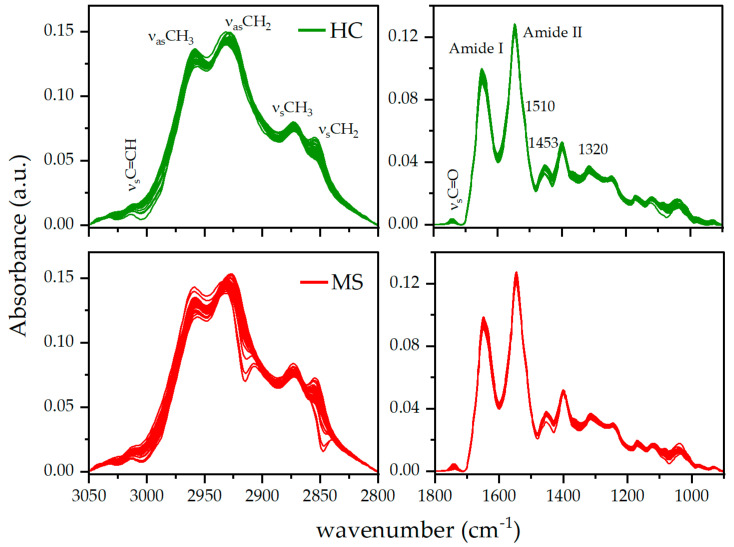
ATR-FTIR spectra of plasma of HC and MS patients in the high region (**left** panels) and fingerprint region (**right** panels). Pre-processing spectral analysis includes cut, rubberband baseline subtraction, and vector normalization. The stretching vibration of specific functional groups and peaks of interest are also shown (see text for details).

Figure 1 shows the ATR-FTIR spectra of MS patients and HC in the two most significant regions: 3050–2800 cm^−1^, containing the symmetric and asymmetric stretching vibrations of CH_2_ and CH_3_, and the fingerprint region in the 1800–900 cm^−1^ range, where the most prominent peaks are the amide I and amide II peaks that are due to the absorption band of proteins. 

The visual inspection of the spectra does not provide any information about key specific features of MS disease. To reveal the differences between the two groups of individuals, we can determine a number of spectral indicators correlated both with the molecular components present in the plasma and with specific peaks associated with molecular functional groups. To this end, in the present analysis, we consider several spectral indicators (see Table 1), some of them previously derived [28] and others determined in the current analysis using the same experimental spectra. Similar indicators were considered in previous studies (see the references in Table 1) and describe (1) the area ratios of molecular components such as lipid/proteins determined as the area, A_HR_, corresponding to the region between 3050 and 2800 cm^−1^ and the area corresponding to the amide I and amide II peak, A_HR_/A_amideI+amideII_, the area of the olefinic C=CH peak over A_HR_, A_olefinic_/A_HR_, the area of the asymmetric stretching vibration peak of CH_2_, A_CH2as_/A_HR_, and A_amide I_/A_amide II_; (2) the intensity ratio of specific peaks of functional groups, I_1453_/I_1650_, I_1739_/I_1468_; (3) the bandwidth of amide I and of the ester C=O peak at 1739 cm^−1^; and (4) the intensity at 1320, 1510, 2860, and 3016 cm^−1^, selected as important wavenumbers for the discrimination of MS from HC according to the random forest classification algorithm. Bandwidth values were determined at 75% of the height of the maximum peak intensity from the baseline. 

**Figure 2:**
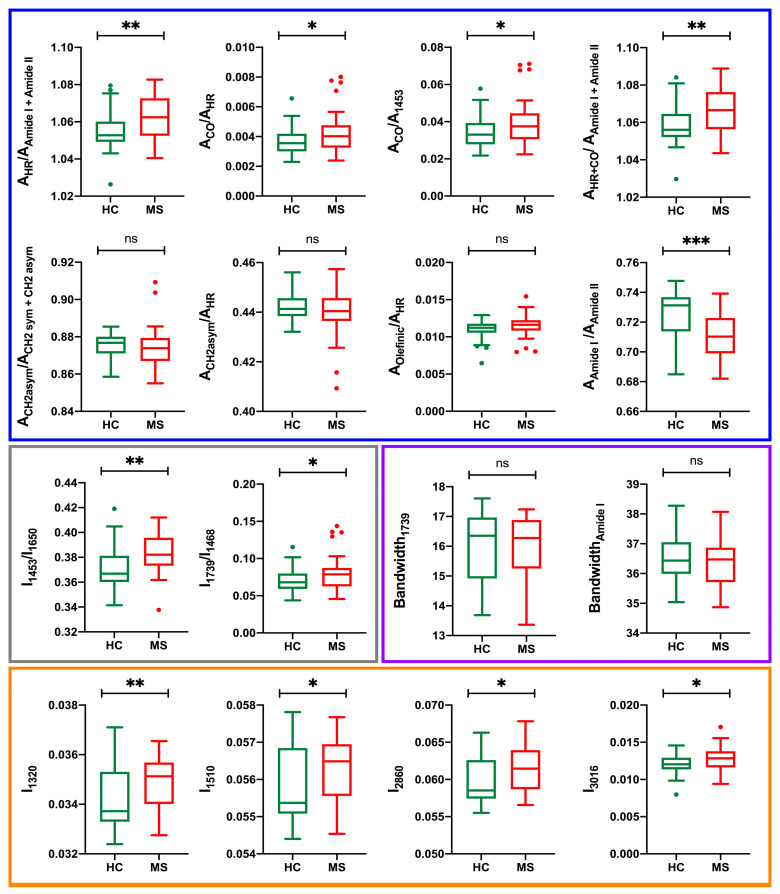
Box-plot of the spectral parameters derived from the analysis of the ATR-FTIR absorption spectra, illustrating the distribution of their values in the HC and MS groups. The *p*-value is indicated as * (*p* ˂ 0.05); ** (*p* ˂ 0.01); *** (*p* ˂ 0.001).

As a preliminary statistical evaluation, we determined the average and standard deviation of these spectral indicators, and assessed the mean differences between MS and HC using two-tailed *t*-test. The *p*-values reported in Table 1 show that most of the spectral parameters have a statistical significance (*p* < 0.05), but for five of them the difference between the two groups of subjects is not significant. In Figure 2 the box-plots of each individual parameter are reported. 

### 3.2. Linear Predictor for Classification

As a further step, our goal was to simultaneously select and combine the most significant spectral indicators described in the above section into a concise predictive tool for diagnostic purposes. In this respect, as described in Section 2.2, we started from the full model containing all the spectral indicators reported in Table 1, and obtained the final model via a backward stepwise selection to remove the irrelevant indicators. The remaining variables, which mainly contributed to the classification of the two groups of subjects, are the six indicators reported in Table 2. In the same table, the fitted coefficients of the linear combination described in Equation (2) are given, together with their standard error and the corresponding results from the Wald test. 

**Figure 3:**
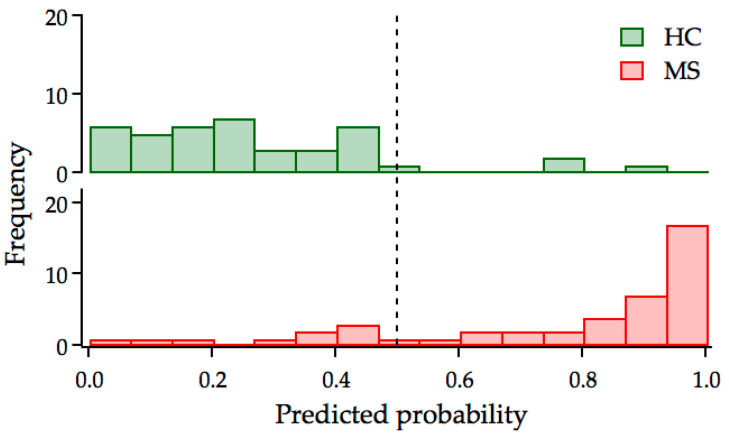
Distribution of the predicted probability for HC (**upper** panel) and MS (**bottom** panel) individuals. The dashed vertical line indicates the 0.5 score threshold. Score values ≤0.5 indicate subjects classified as healthy, score values ˃0.5 indicate subjects classified as diseased.

Considering the obtained values for the selected indicators, a classification for each subject can be obtained and compared with their real health status, to assess the capability of the model in distinguishing between patients and controls. A comparison of the predicted probability distributions within the HC and MS groups is shown in Figure 3. The plot clearly shows a very good performance of the model, with the clustering of the HC subjects on the left and the MS on the right of the cutoff dashed line at 0.5. The misclassified subjects in both groups are only a few samples and these are located, respectively, to the right and to the left of the threshold value.

**Figure 4:**
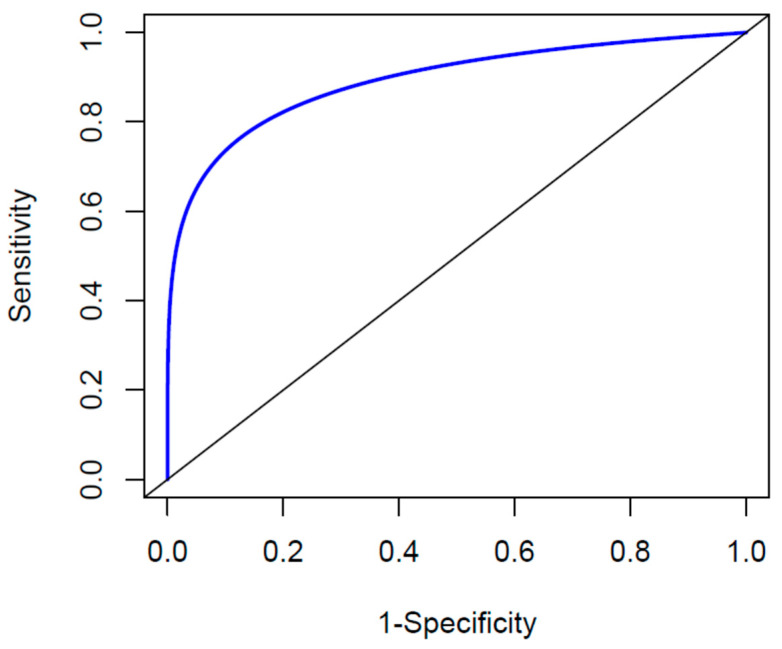
Predictive power of the obtained model in distinguishing between HC and MS, as described by a smoothed ROC curve.

In Table 3, the values for sensitivity, specificity, accuracy, and AUC are reported for both the whole MS group and separated in subgroups according to EDSS values or disease duration. In all cases, the indicators show the good capability of the fitted model to distinguish between MS and HC, with an accuracy and an AUC higher, respectively, than 85% and 87%. The accuracy of the model is also reflected in the global ROC curve, which is characterized by an AUC value of 0.9 (Figure 4). Moreover, no difference in the AUC of the ROC curves is found when patients with different EDSS scores (*p*-value = 0.833) or disease duration (*p* = 0.265) are compared.

Tests of the statistical significance of the classification score values were performed in the HC and MS groups according to age and gender. No significant correlation was found between the linear predictor values and age in the MS (*p*-value = 0.521) or control group (*p*-value = 0.610), and no difference in the scores’ distribution was observed between males and females (MS: *p*-value = 0.983; HC: *p*-value = 0.529). 

## 4. Discussion

The diagnosis of MS, and in general of neurodegenerative diseases, may be very complex, as there is no clinically validated single test available. The need for new diagnostic tools based on advanced experimental and computational techniques could be of considerable support to neurologists, and allow patients to be treated early with therapies to control these currently incurable diseases. In particular, biofluid spectroscopy is relevant in the field of liquid biopsy for searching and identifying diagnostic biomarkers, with the additional support of multivariate analysis [17,33].

For our investigation, we used ATR-FTIR spectroscopic data obtained from plasma samples collected during the routine clinical observation of MS patients. The FTIR absorbance spectra recorded for the diseased subjects showed similar spectral features compared to those registered for the healthy subjects, both in the high region (3100–2800 cm^−1^) and in the fingerprint region (1800–900 cm^−1^) of the spectra. This similarity has been found in other FTIR studies on plasma [19,20,30], and accounts for the similarity of the molecular components. To highlight subtle spectral differences between the two classes of subjects, several indicators related to the two most representative molecular components, lipids and proteins, were selected. Almost all of these bioindicators (Table 1) show statistical significance, reveal specific biochemical changes, and, therefore, are good candidates for sample differentiation. In particular, the lipid/protein ratio in the MS group is higher than the HC group’s. The increase of the lipid level is observed in the plasma of MS patients and, in parallel, the protein level decreases, as it can be evidenced, for example, by the A_amide I_/A_amide II_ ratio. The trend of these molecular components is an important signature for MS disease and depends on the biofluids interrogated. In fact, our results agree very well with previous FTIR studies on the serum of MS patients, where a total protein content decrease and a lipid content increase, with respect to controls, was also observed [30]. On the contrary, in the CSF this ratio is reversed [29]. The altered lipid content is a direct consequence of the demyelination process that is a hallmark of MS. Within the lipid analysis it can be also noted that the intensity of the C=O band increases in MS, possibly due to the presence of oxidized lipids produced by free radicals whose concentration increases during inflammatory states [34,35]. Similarly, concerning the protein content in plasma, where albumin and immunoglobulin G (IgG) are the two most abundant proteins, the observed decrease may result from the extravasation of plasma components through damaged vessels. Interestingly, the albumin and IgG quotients, related to their concentration in the serum and CSF, are a measure of the blood–CSF dysfunction and have been used as an indicator of blood–brain barrier (BBB) integrity [36,37].

In addition to the univariate analysis of the spectral parameters that can potentially be linked to disease-induced biochemical changes, we combined these indicators to construct a new diagnostic index. The result of this procedure is a linear predictor in which only the spectral parameters that jointly contribute most to the sample differentiation of the two classes, HC and MS, are included (Table 2). The six spectral biomarkers that we established in the final model belong to both the infrared regions considered, the high region and the fingerprint region. It is interesting to note that the regression analysis reveals the significant role of the A_CH2asym_/A_CH2sym+CH2asym_ ratio in predicting the healthy status, even if the mean difference between HC and MS patients appears not to be significant in the univariate analysis. This is due to the correlation between the considered covariates that, even if it does not pose a multicollinearity problem, acts in determining a suppressor effect (see, for example, [38,39]). This simple predictive tool provides, for our cohort of samples, a better classification in terms of sensitivity, specificity, and accuracy compared to the previous analysis based on random forest, PCA-LDA, and PLS-DA algorithms [28]. In fact, sensitivity increased from 78 to 80% whereas specificity increased from 83 to 92.5%, and the AUC value was 0.9. Compared with the research data on the spectroscopic analysis of biological samples for MS and other neurological diseases, the performance values obtained for our model are, as far as we know, the most favorable. In fact, in MS studies, an AUC value of 0.86 was obtained in a FTIR study on CSF aiming to separate patients with clinically isolated syndrome from RRMS patients [29], whereas slightly lower AUC values (0.82–0.83) were obtained from NMR data [40]. Finally, by using the random forest model on FTIR fingerprint data, an overall precision of 83.3% was found when distinguishing healthy from pathological (MS and amyotrophic lateral sclerosis) serum samples [41]. Considering other neurological diseases, such as Alzheimer’s, Paraskevaidi et al. [25] reported specificity and sensitivity values of 70% from the FTIR blood analysis of the fingerprint region. These values increased to 86% when other clinical data were included in the classification model. A high level of performance (˃90%) for classification models have been generally reported for the spectroscopic analysis of biological fluids (plasma, serum, saliva) for cancers [14,18,19] and COVID-19 [20,22] detection. Obviously, when the biofluid used is in contact with the pathological region, it is more sensitive in reporting disease-related information. However, peripheral blood and its constituents seem to be the most convenient specimens for diagnostic and biomarker detection. In fact, the challenge with liquid biopsy is the possibility of using readily available biofluids with little or no invasiveness for the patients. 

## 5. Conclusions

The results presented in this study have demonstrated that ATR-FTIR spectroscopy applied to human plasma, supported by statistical regression methods, provides valuable spectral biomarkers that can be used for further analysis to improve sample classification in MS disease. A set of parameters was derived from univariate and multivariate statistical analysis of the experimental infrared spectra representing the biochemical fingerprint of the biofluid of HC and MS patients. These spectral biomarkers, characterized by a different statistical significance value, were combined into a linear predictor representing a single, simple diagnostic index whose value varied between 0 and 1. Through a backward stepwise procedure, the irrelevant variables were removed, and only the six most important ones were considered in the final model. Using this model, the predicted probability calculated for our samples provides a better performance than that previously obtained by PCA-LDA, PLS-DA, and RF classification algorithms. It is important to point out that the two approaches are not alternative and, in fact, this new data analysis used spectral biomarker data partially derived in previous work. 

To create a more general validity, the logistic regression model should be applied to different infrared spectra for sampling other diseases, in which a sub-optimal level of classification has been obtained. Moreover, it should be also made clear that the starting set of parameters may be different from the ones we have used here and should be related to the data set available and to the possible biochemical differences of the disease. 

Finally, the study we presented, consisting of human plasma analysis with ATR-FTIR spectroscopy, combined with suitable data analysis, is a very promising diagnostic tool for MS, demonstrating that even for neurological diseases it is possible to obtain very effective discrimination results.

## Figures and Tables

**Table 1:** Spectral indicators derived from data in Figure 1, indicating the area ratios of the lipid/protein components, intensity ratio of specific peaks; bandwidth of the ester C=O at 1739 cm^−1^ and of the amide I peak; intensity values in the fingerprint region (1320 and 1510 cm^−1^) and in the high region (2860 and 3016 cm^−1^). Values are given as average ± standard deviation. The *p*-value is obtained via unpaired *t*-test. The last column refers to studies with a similar choice of the indicated spectral regions and wavenumber of functional groups.

	HC	MS	*p*-Value	Refs
A_HR_/A_amide I + amide II_	1.0552 ± 0.0107	1.0621 ± 0.0114	0.005	[25,28,31]
A_C=O_/A_HR_	0.0036 ± 0.0009	0.0042 ± 0.0013	0.028	[29]
A_C=O_/A_1453_	0.0341 ± 0.0080	0.0388 ± 0.0120	0.038	[28]
A_HR+C=O_/A_amide I + amide II_	1.0591 ± 0.0112	1.0666 ± 0.0122	0.004	[29]
A_CH2asym_/A_CH2sym + CH2asym_	0.8750 ± 0.0068	0.8744 ± 0.0101	0.776	[31]
A_CH2asym_/A_HR_	0.4423 ± 0.0059	0.4402 ± 0.0097	0.230	[29]
A_Olefinic_/A_HR_	0.0110 ± 0.0012	0.0115 ± 0.0015	0.106	[29]
A_amide I_/A_amide II_	0.7250 ± 0.0165	0.7115 ± 0.0136	<0.001	[29]
I_1453_/I_1650_	0.3719 ± 0.0166	0.3834 ± 0.0148	0.001	[25,28]
I_1739_/I_1468_	0.0702 ± 0.0158	0.0796 ± 0.0229	0.034	[28]
Bandwidth_C=O_	16.0404 ± 1.1338	15.9344 ± 1.0337	0.653	[28]
Bandwidth_amide I_	36.5558 ± 0.7052	36.3905 ± 0.7795	0.310	[28]
I_1320_	0.0342 ± 0.0012	0.0349 ± 0.0010	0.003	[28]
I_1510_	0.0559 ± 0.0010	0.0563 ± 0.0008	0.024	[25,28]
I_2860_	0.0597 ± 0.0030	0.0616 ± 0.0031	0.004	[28]
I_3016_	0.0120 ± 0.0013	0.0128 ± 0.0016	0.016	[28]

**Table 2:** Fitted coefficients ${\hat{\beta }}_{j}$ for the indicators in the final model, corresponding standard errors, and results from the Wald test.

	${\hat{\beta }}_{j}$	S.E.	z	*p*-Value
A_CH2asym_/A_CH2sym + CH2asym_	659.218	177.363	13.814	<0.001
A_amide I_/A_amide II_	−200.233	53.352	14.086	<0.001
I_1453_/I_1650_	58.460	24.677	5.612	0.018
I_1320_	−1897.473	710.464	7.133	0.008
I_1510_	−2128.135	670.065	9.821	0.002
I_2860_	1618.604	498.808	19.530	0.001
Constant	−367.473	139.994	6.890	0.009

**Table 3:** Performance of the fitted linear predictor in distinguishing HC from MS, considered either as a whole group or divided in subgroups according to disability level and disease duration.

		Sensitivity (%)	Specificity (%)	Accuracy (%)	AUC
		80.00	92.50	85.88	0.90
EDSS	0.5–3.0	81.25	92.50	87.50	0.90
	3.5–7.0	76.90	92.50	88.70	0.89
Disease Duration	≤10y	81.50	92.50	88.10	0.87
	>10y	77.70	92.50	87.90	0.94

## Data Availability

Additional raw data are available from the corresponding author upon reasonable request.
